# Review on Wood Deformation and Cracking during Moisture Loss

**DOI:** 10.3390/polym15153295

**Published:** 2023-08-03

**Authors:** Zongying Fu, Jiaxing Chen, Yongyue Zhang, Feifan Xie, Yun Lu

**Affiliations:** 1 Key Laboratory of Wood Science and Technology, State Forestry and Grassland Administration, Institute of Wood Industry, Chinese Academy of Forestry, Beijing 100091, China; zyfu@caf.ac.cn (Z.F.); ff66997757@163.com (F.X.); 2 School of Material Science and Art Design, Inner Mongolia Agricultural University, Hohhot 010018, China; cxx18547412993@163.com; 3 School of Materials Science and Engineering, Nanjing Forestry University, Nanjing 210018, China; 18195486182@163.com

**Keywords:** wood, moisture content, mechanical property, deformation and cracking

## Abstract

Wood, being a natural hygroscopic material, the interaction between wood and moisture plays a crucial role in wood processing and utilization. Moisture affects the physical and mechanical properties of wood, and is also one of the main external factors that cause wood deformation and cracking. Drying shrinkage is a common phenomenon during the processing and utilization of wood induced by moisture loss. Drying stress is the main cause of wood deformation and cracking. The shrinkage differential between tangential and radial direction and moisture content gradient of wood are two reasons induced the generation of drying stresses. In this review, the existing states of moisture in wood and the interaction between water molecules and wood components were systematically summarized. The current research progress and deficiencies in three aspects including the factors resulted in deformation and cracking in wood caused by moisture loss, the correlation between wood mechanical properties and moisture, as well as the development of deformation and cracking in wood under moisture loss were discussed. This review aims to facilitate further research on the deformation and cracking of wood under moisture loss by providing valuable insights and assistance, ultimately reducing the occurrence of wood deformation and cracking. And thus, it will enhance the overall utilization of wood resources, making wood better serve human life.

## 1. Introduction

**Figure 1:**
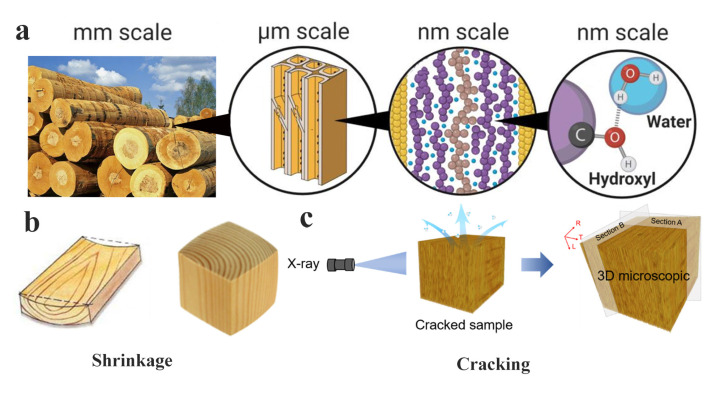
Relationship between wood and moisture. (**a**) Microstructure of wood at different scales and its interaction with water [2], (**b**) Wood shrinkage [21], and (**c**) drying cracking [22]. (**a**) Reprinted/adapted with permission from Ref. [2]. 2021, the authors. Licensee MDPI. (**b**) Reprinted/adapted with permission from Ref. [21]. 2022, the authors.

Wood, as one of natural macromolecular material directly derived from nature, has the characteristics of low production cost, low energy consumption, non-toxicity, and non-pollution. Compared with steel, cement, plastic, wood is also a renewable, degradable, and recyclable green material [1]. As shown in Figure 1a, the vitality of trees is intrinsically linked to moisture, and the removal of moisture from the wood to a specific target level is a necessary process for wood processing and utilization. The interaction between wood and moisture is the core of wood processing and utilization [2]. Moisture directly affects the physical and mechanical properties, durability, and dimensional stability of wood [3,4,5,6,7,8]. The moisture content in wood is defined as the amount of water present in the wood, expressed as a percentage of its dry weight. The moisture content of wood can range from 0% (completely dry) to over 200% (more water weight than wood substance). The fiber saturation point in drying wood at which all free moisture has been removed from the cell itself while the cell wall remains saturated with absorbed moisture.

Drying shrinkage of wood is one of the most representative reflection of the interaction between wood and moisture [9]. As shown in Figure 1b, drying shrinkage refers to the reduction in the dimensions of wood that occurs, when wood moisture below the fiber saturation point. Wood is also known as an anisotropic material, it stems from its biological composition. Its unique physical properties are based on the structure of the wood cells and how they are arranged. In wood, the anisotropic nature is often seen in three perpendicular directions: axial (along the grain or trunk of the tree), radial (from the center of the tree to the bark), and tangential (perpendicular to the grain and tangentially to the growth rings). This anisotropy affects many of the wood’s characteristics such as strength, shrinkage, swelling, thermal and electrical conductivity. As a representative example, wood exhibits different levels of shrinkage among tangential, radial and longitudinal directions. Generally, the tangential shrinkage is the greatest, around 6–12%, followed by radial shrinkage at 3–6%, and longitudinal shrinkage being less than 0.1–0.2% [10]. The loss of moisture leads to different drying shrinkage in different grain directions of wood, and thus shrinkage anisotropy of wood is one of the reasons for the drying stresses. Additionally, due to inconsistencies of moisture content between the surface and core layers during the drying process of wood, as well as differences in material properties between heartwood and sapwood, or earlywood and latewood, there will be uneven distribution of moisture content. The uneven distribution of wood moisture will produce moisture gradient stress, which forms an additional source of drying stresses in wood. Therefore, the shrinkage anisotropy stress and moisture gradient stress, are the two principal catalysts of drying stresses in wood [11].

Drying stresses are the internal stresses in the process of wood drying, and acting as a mutual restriction within the internal tissues of the wood. It is closely related to internal factors such as the wood component, material properties, and moisture content, as well as external environmental factors such as drying temperature and relative humidity [12,13,14,15]. Generally, in the early stage of drying, the drying stresses are small and will not damage the wood. However, below the fiber saturation point, as moisture content decrease, the drying stresses gradually increase. When the drying stresses exceeds the limit of the tensile strength in wood tangential direction, drying cracking will occur [16], as shown in Figure 1c. Cracking usually originates at the parts with large differences in drying shrinkage, such as the junction between heartwood and sapwood, as well as the transition zones between earlywood and latewood. Moreover, the initiation of cracking is also affected by tree species.

Apart from drying stresses, the mechanical properties of the wood also affects the deformation and cracking of wood and are also closely related to moisture content [17]. Generally, when the moisture content is below the fiber saturation point, the strength of the wood decreases as increasing moisture content. This is due to the increase of bound water gradually softens wood cell wall [18,19,20]. However, when the moisture content of wood is above the fiber saturation point, the strength and mechanical properties are independent of moisture content. The moisture content has a great impact on the compressive and bending strength parallel to grain of the wood, but almost no impact on the tensile strength parallel to grain direction.

In this review, the existing states of moisture in wood and the interaction between water molecules and wood components were systematically summarized. The current research progress and deficiencies in three aspects including the factors resulted in deformation and cracking in wood caused by moisture loss, the correlation between wood mechanical properties and moisture, as well as the development of deformation and cracking in wood under moisture loss were discussed. This review aims to facilitate further research on the deformation and cracking of wood under moisture loss by providing valuable insights and assistance, ultimately reducing the occurrence of wood deformation and cracking and enhancing the overall utilization of wood resources.

## 2. Moisture in Wood

### 2.1. Existing State of Moisture in Wood

Water in wood can exist in three main forms: free water, bound water, and water vapor [23]. Free water exists in large capillary system of wood in free state, such as cell cavities, intercellular spaces, and vessel lumina and pit cavities. Free water is physically associated with the wood but not tightly bound, allowing it to lose easily from wood. Bound water exists in the microcapillary system such as the gaps between microfibrils within the cell wall, or it can be adsorbed onto the free hydroxyl groups on the surface of cellulose molecules in the amorphous regions. Bound water is closely combined with wood and is not easy to escape from wood. It can only evaporate from the wood when all the free water within the wood has completely evaporated, and the vapor pressure of water in the wood exceeds that of the surrounding environment. When the moisture content in wood changes above the fiber saturation point, it primarily affects the quantity of free water, with minimal impact on wood properties. Conversely, when the moisture content in wood changes below the fiber saturation point where free water is absent, variations in bound water has a great impact on wood properties. The mechanical properties, shrinkage, swelling, thermal conductivity, and electrical conductivity, all change with the variation in bound water. Consequently, the quantity of bound water is considered a major factor influencing wood properties, and thus much attention should be paid to the changes in bound water during wood processing.

### 2.2. Interaction between Water Molecule and Wood Components

**Figure 2:**
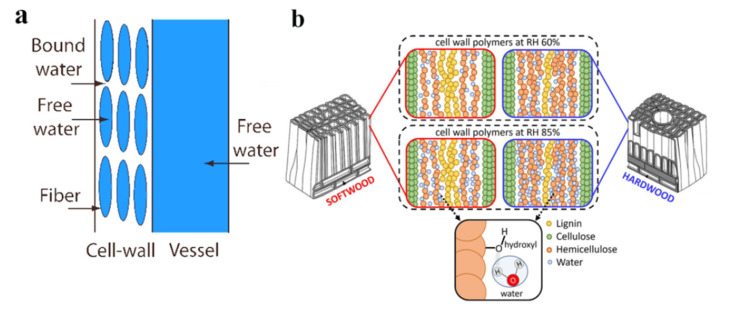
(**a**) The presence of moisture in wood [24], (**b**) Interaction of water molecules with cellulose, hemicellulose and lignin [25]. (b) Reprinted/adapted with permission from Ref. [25]. 2022, The Authors.

The presence of moisture in wood and interaction of water molecules with cellulose, hemicellulose and lignin, as shown in Figure 2a,b, the interaction between wood and moisture primarily involves non-covalent bonding between the molecules of water and cellulose, hemicellulose, and lignin. Water molecules are not absorbed into the crystalline region because of close packing of the structure, it always bind to the hydroxyl groups in the amorphous region of cellulose molecular chains, and thus the binding of water molecule with cellulose is closely related to cellulose crystallinity. When water molecules bind to these hydroxyl groups, adjacent cellulose chains will be separated, leading to the expansion of fibers. In comparison with cellulose, hemicellulose possesses a higher number of hydroxyl adsorption sites, thereby exhibiting a stronger ability to bind with water. Through the formation of hydrogen bonds, water molecules can fill the space within the hemicellulose matrix and the interfaces between cellulose and hemicellulose molecules, consequently causing the swelling of wood cell walls. Lignin contains hydroxyl, carbonyl, and methoxy functional groups, which possess hydrophilic characteristics. However, due to the presence of phenyl in its basic chain units, lignin exhibits poor compatibility with water. Nevertheless, in the presence of moisture, water molecules disrupt the hydrogen bonds between lignin molecules and establish separate hydrogen bonds with any available hydroxyl group sites in lignin.

Moreover, water molecules have an impact on the glass transition temperature (Tg) of wood through interacting with lignin. Water molecules tend to bind with lignin, lowering the Tg, making the wood more pliable. When wood dries out, these water molecules are removed, leading to an increase in Tg and in the stiffness of the wood. This is also why wood’s mechanical properties change with varying humidity. Moreover, this relationship between water content and Tg plays a crucial role in various wood processing and use scenarios such as drying, thermal modification, and mechanical deformation.

## 3. Causes of Wood Deformation and Cracking during Drying

Wood, as a hygroscopic material, meaning it attracts and holds water molecules from the surrounding environment, and this process is termed as adsorption. In contrast, when the environmental conditions change, or when wood is heated, the water molecules move from the wood back into the environment, which is known as desorption. During the initial phase of timber drying, which involves the transformation from a log to a stable board, predominant effects include shrinkage and collapse. The stable board’s response to changing moisture content conditions when in service may involve shrinkage or expansion. Either scenario could potentially lead to distortions such as peaking and cupping. Here, the deformation and cracking is only discussed during moisture loss of wood.

Drying shrinkage is a common phenomenon during desorption, which is a distinctive trait that closely related to the moisture content below the fiber saturation point. The drying defects in wood during the drying process is primarily attributed to drying stress. If we exclude the influence of inherent growth stress in wood, drying stress mainly arises from the shrinkage anisotropy and moisture content gradients. The two factors serve as the primary drivers of wood deformation and cracking.

### 3.1. Shrinkage Anisotropy Stress

**Figure 3:**
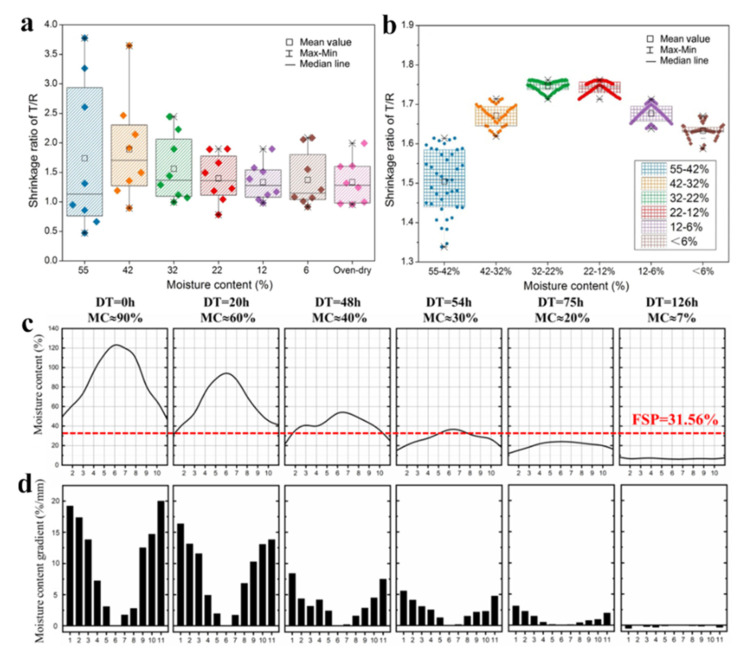
Anisotropic shrinkage and moisture gradients in wood. Anisotropic shrinkage between the tangential and radial direction in the cellular (**a**) and macroscopic level (**b**) [52]; Layer moisture content and moisture content gradient of sawn timber in drying process (**c**,**d**). (**a**,**b**) Reprinted/adapted with permission from Ref. [52]. 2021, Taylor & Francis Group, LLC.

The differential shrinkage between the tangential and radial directions during the drying process is one factor contributing to wood deformation and cracking. As shown in Figure 3a,b, the tangential and radial shrinkage of wood is determined by its inherent properties and plays a crucial role in the generation of drying stresses during moisture loss [26]. Researchers such as Barber et al. have attributed the anisotropic shrinkage in wood to the alignment of microfibrils within the cellulose crystalline regions of the cell wall, and demonstrated this influence of microfibrils orientation on shrinkage through modeling [27]. Additionally, studies by Cave, Koponen, and Yamamoto have further elucidated the anisotropic shrinkage of wood by describing the effects of microfibril angles on shrinkage behavior using various models [28,29,30,31]. Yamashita et al. investigated the diversity of tangential and radial shrinkage in Japanese cedar (*Cryptomeria japonica*) wood. The results showed that microfibril angles were the primary factor affecting tangential shrinkage, while factors such as basic density, annual ring width, and latewood percentage mainly influenced radial shrinkage [32].

Additionally, there are variations in anisotropic shrinkage stress along the pith-to-bark direction. Fu et al. investigated the shrinkage characteristics of white birch (*Betula platyphylla*) wood under shrinkage anisotropy stress using cross-section discs. Results revealed that the tangential shrinkage ratio was 1.12 to 1.54 times greater than that of radial shrinkage. Differences in shrinkage characteristics were observed between heartwood and sapwood, and the differential shrinkage ratio between the tangential and radial directions increased with decreasing moisture content [33,34]. Gao et al. conducted testing and analysis of shrinkage in Masson pine (*Pinus massoniana*) wood using digital image correlation with VIC-3D system, which reported a tangential shrinkage ratio of 5.5% and a radial shrinkage ratio of 3.5% [35]. In summary, the magnitude of differential shrinkage between the tangential and radial directions during wood drying will directly affect the drying quality of wood. Larger differences in tangential and radial shrinkage at the same moisture content result in higher levels of shrinkage anisotropy stress and a greater likelihood of drying defects in wood.

### 3.2. Moisture Content Gradient Stress

Moisture content gradient stress is another factor contributing to wood deformation and cracking, is commonly observed during wood drying process. As shown in Figure 3c,d, in green wood, softwood species typically exhibit higher moisture content in the sapwood region compared to the heartwood region, while variations exist among hardwood species [36]. Extensive research has been conducted on moisture content gradient stress during the drying process. The effect of moisture gradient stress on drying cracking during drying process of European Spruce (*Picea abies*) cross-section disc is shown as follows: there is moisture content gradients both in longitudinal and tangential directions in the cross-section discs. The influence of longitudinal moisture content gradient on disc cracking behavior varied with thickness, with a more pronounced effect observed in discs thicker than 30 mm, while no evident effect was observed in discs thinner than 15 mm [37,38].

Different from wood cross-section discs, the movement and evaporation rate between wood surface and interior during lumber drying are differential, and thus resulting in moisture content gradients between the core and surface layers. The drying stresses generated by moisture content gradients are the primary cause of drying cracks in lumber. There has yielded relatively mature theories on the research of drying stresses during lumber drying, which can be summarized as follows: during the drying of lumber, the moisture content of the surface layer is lower that of the core layer, reaching a moisture content below the fiber saturation point and initiating shrinkage. At this stage, the moisture content in the core layer remains above the fiber saturation point, and the shrinkage of the surface layer is restrained by the core layer, leading to tensile stress in the surface layer and compressive stress in the core layer. As drying continues, the moisture content in the core layer decreases below the fiber saturation point, it also undergoes shrinkage. However, due to the plastic deformation that occurred in the surface layer under previous tensile stress, the normal shrinkage of the core layer is counteracted, resulting in tensile stress in the core layer and compressive stress in the surface layer [39].

Experimental testing and simulation prediction are the main approaches used in studying moisture content gradients. Experimental testing methods include the slicing method [40], CT scanning [41], X-ray microscopy [42], X-ray profile density method [43,44], low-field nuclear magnetic resonance [45,46,47], and others. In terms of simulation model, it can be dated back to the early 1980s. Pang et al. developed a two-dimensional model to predict the moisture content distribution during the drying process of radiata pine (*Pinus radiata*) wood. This model was validated through CT scanning experiments, demonstrating its accurate prediction of moisture content distribution across the thickness, width, and growth ring direction of the lumber [48,49]. Haquea constructed a two-dimensional drying model to simulate the moisture content gradient within radiata pine (*Pinus radiata*) lumber during high-temperature drying, and used various experimental methods to investigate the moisture content gradient during the wood drying process [50]. Moreover, for square lumber drying, Yamashita et al. found that larger cross-sectional dimensions were associated with more severe surface cracking in Japanese cedar (*Cryptomeria japonica*) lumber. This was attributed to the greater moisture content gradient between the heartwood and sapwood regions in larger cross-sectional dimensions of the lumber [51].

## 4. Relationship between Wood Mechanical Properties and Moisture Content

The occurrence of wood deformation and cracking requires the drying stress to exceed the ultimate tensile strength in wood tangential direction. While the drying stress remains constant, the mechanical properties of wood play a decisive role in determining the occurrence of deformation and cracking. The elastic modulus, strength and other mechanical properties of wood exhibit a close relationship between wood moisture content. Therefore, the interplay between wood mechanical properties and moisture content has an important impact on the occurrence of wood deformation and cracking.

### 4.1. Relationship between Wood Transverse Mechanical Properties and Moisture Content

Research on the relationship between wood mechanical properties and moisture content primarily focuses on the longitudinal direction, with relatively less emphasis on the transverse direction. It is generally believed that above the fiber saturation point, moisture content has little influence on wood mechanical properties. However, below the fiber saturation point, as moisture content decreases, the elastic modulus, strength, and other mechanical properties tend to increase. The transverse tensile strength of wood (perpendicular to the grain direction) is much smaller than the longitudinal tensile strength (parallel to the grain direction). The cracking of wood during drying occurs when the drying stress exceeds the ultimate tensile strength of wood tangential direction. Therefore, the transverse tensile strength of wood determines its deformation and cracking of wood during moisture loss.

The study conducted by Ozyhar et al. focused on investigating the relationship between transverse mechanical properties and moisture content in European beech (*Fagus sylvatica*). They performed uniaxial tensile tests on samples with varying moisture content levels ranging from 7.8% to 16.9%. The results of the study revealed that both the tensile modulus of elasticity and tensile strength decreased as the moisture content of the European beech samples increased. Specifically, the mechanical properties experienced a decrease of approximately 2–3% for every 1% increase in moisture content [53]. The study conducted by Jiang et al. involved investigating the elastic and strength parameters of Japanese cedar (*Cryptomeria japonica*) wood in different moisture content conditions. They examined the wood’s properties in three principal axes under four moisture content levels: 10.3%, 12.2%, 14.6%, and 16.7%. Their findings were consistent with the previous study by Ozyhar et al. Specifically, the tensile modulus of elasticity in the tangential direction decreased from 8.1 GPa at 10.3% moisture content to 4.5 GPa at 16.7% moisture content [54]. In addition, Miyoshi et al. conducted experimental tests on the transverse tensile strength of ten different wood species. They found that this strength parameter is influenced by various factors such as cell type, cell shape, arrangement, and the degree of earlywood-latewood transition [55].

The study conducted by Zhan et al. examined the influence of moisture content on the transverse tensile strength of two different wood species: Poplar (*Populus* sp.) and Chinese fir (*Cunninghamia lanceolata*) [56]. They found that under the same moisture content conditions, the radial tensile strength (perpendicular to the growth rings) was higher than the tangential tensile strength (parallel to the growth rings). Furthermore, the results indicated that an increase in moisture content led to a decrease in transverse tensile strength. Specifically, when the moisture content increased from 0% to the fiber saturation point, the tangential tensile strength of Poplar (*Populus* sp.) decreased by 20.3%, and that of Chinese fir (*Cunninghamia lanceolata*) decreased by 34.8%. In another study by Yue et al., they suggested that the transverse tensile strength and tensile modulus of elasticity of Chinese fir (*Cunninghamia lanceolata*) exhibited a linear decrease with increasing moisture content within the range of 7% to 20% [57].

### 4.2. Relationship between Mechanical Properties of Wood Cell Wall and Moisture Content

The specific relationship between macroscopic properties of wood and moisture content is largely determined by the microstructure of wood. However, there is currently a limited amount of research available on the connection between the mechanical properties of wood cell walls and moisture content. Yamamoto et al. conducted a study on the relationship between cell wall components and the longitudinal elastic modulus in Japanese cedar (*Cryptomeria japonica*). They introduced a simplified model of wood fibers, enabling a theoretical representation of the connection between longitudinal elastic modulus and moisture content. They suggested that the impact of moisture content on the elastic modulus is attributed to the presence of a transition zone between the crystalline and amorphous regions of cellulose microfibrils. This transition zone undergoes fluctuations within this transition zone as moisture content changing, thereby influencing the mechanical properties of wood cell walls [58,59].

**Figure 4:**
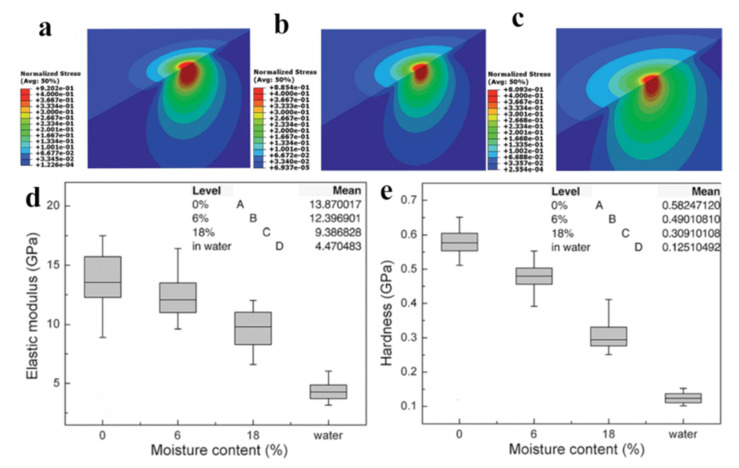
Effect of moisture content on wood mechanical properties. Correlate the moisture content with the cell wall’s ability to creep (**a**–**c**); Effect of moisture content on elastic modulus (**d**) and hardness of wood cell walls (**e**) [61]. Reprinted/adapted with permission from Ref. [61]. 2015, The Royal Society of Chemistry.

Yu et al. applied nanoindentation technology to investigate the relationship between the longitudinal mechanical properties of latewood cell walls in Masson pine sapwood and their moisture content. The results highlighted a linear relationship between the elastic modulus, hardness, and compressive yield stress of the wood cell walls, and the moisture content within the examined range. Specifically, it was observed that as the moisture content rose from 4.5% to 13.1%, there was a corresponding decrease in the elastic modulus of the wood cell walls from 20.4 GPa to 16.9 GPa. Moreover, the impact of the moisture content on the hardness was found to exceed its effect on the elastic modulus [60]. As shown in Figure 4a–e, Meng et al. studied the viscoelastic behavior of latewood cell walls in loblolly pine (*Pinus taeda*) at a range of moisture content levels. The results suggested that both the elastic and viscosity constants of the wood decreased with increasing moisture content. They further proposed that increased moisture rendered the wood cell walls softer and thicker. This induced greater susceptibility to disruption in the hydrogen bonds among microfibrils, culminating in a decrement in the mechanical properties of wood [61].

## 5. Study on the Law of Wood Deformation and Cracking

### 5.1. Study on Cracking in Macroscopic Scale

In the field of wood macroscopic cracking, Wahl et al. employed a method utilizing laser reflection intensity to examine surface microcracks in wood. The reflective intensity of the wood surface was used as a measure to characterize the state of the cracking [62]. Utilizing this method, Hanhijärvi et al. undertook an exhaustive investigation into the formation and progression of micro-cracks on the wooden surface throughout the drying process. They found that these micro-cracks emerge on the wood surface during the initial stage of drying. As the drying process continues, these micro-cracks slowly close up, but not before aiding in the development of macro-cracks during the later stages of drying [63]. The release and reversal of drying stresses promote the closure of the crack. Yamashita et al. conducted a study on both surface and internal cracking during the drying process of Japanese cedar (*Cryptomeria japonica*) square timber. Their findings indicated that the ratio of heartwood and radial shrinkage primarily influence surface cracking. Specifically, the length of the cracks displayed a direct proportionality to the radial shrinkage. Moreover, internal cracking near the pith was found to be relatively severe, largely due to the impact of radial shrinkage [64,65]. Phonetip et al. compared two internal check measurement methods for wood drying quality assessment [66]. Larsen et al. studied the types of cracks and the factors influencing them during the drying process of cross-section wood discs. They determined that cracking generally transpires in the initial drying stage, when the moisture content falls below the fiber saturation point, predominantly along the radial direction. The main factors influencing cracking include the disparity in moisture content between the heartwood and sapwood, the thickness of test specimens, and the differential between tangential and radial shrinkage [67].

Compared to macroscopic cracks, micro-cracks present greater observational challenges due to their scale during formation and expansion. Fan et al. successfully designed a microscopic image acquisition system by ingeniously integrating a microscope with a custom-made drying oven [68]. This innovative system was employed for the drying of Larix gmelinii (*Gmelin larch*) at a temperature of 60 °C, thereby facilitating the capture and tracking of the inception and expansion of microscopic cracks on the wood surface. Sakagami investigated the timing and location of microcrack formation using laser confocal microscope by placing Japanese cedar (*Cryptomeria japonica*) in an environmental control box at 50 °C with a Relative Humidity of 5% [69]. The results showed that as soon as the drying process started, microcracks immediately appeared in the latewood of the heartwood area. The sapwood was the last place where microcracks appeared, and concurrently, the microcracks in the heartwood began to close. Furthermore, most of the microcracks were closed and some had even disappeared completely by the end of the drying process. An experimental measurement device developed by Botter-Kuisc, which can real-time monitor the average moisture content, moisture gradient, and the number of cracks in the wood [70]. This apparatus allows for quantifying the relationship between wood shrinkage differences, moisture gradient, and wood cracks.

### 5.2. Study on Cracking in Cellular Scale

**Figure 5:**
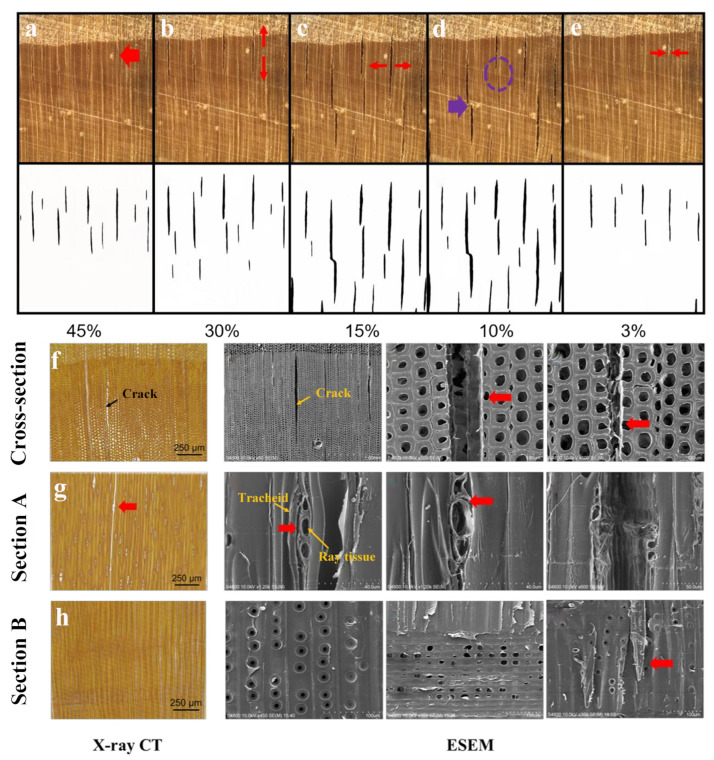
Generation and propagation of drying cracking. Macroscopic cracking at different moisture contents (**a**–**e**); fracture morphologies of samples: X-ray CT images and ESEM images in three anatomy directions (**f**–**h**) [22].

In a study focusing on cracking at the cellular level in wood, Wang et al. conducted an examination of the microscopic cracking patterns in two types of wood during the drying process: Red Oak (*Quercus rubra*) and Long-fruited Oak (*Cyclobalanopsis longinux*). Results showed that wood with a high density and low porosity was more susceptible to cracking, and thick-walled cells were more prone to cracking than thin walled. Multiple initial failure points were discovered in both types of wood, a phenomenon tied to their intricate anatomical structures [71]. Saka et al. proposed that ray cells were more susceptible to cracking, the lignin content in the cell wall of ray cells was lower than that in fiber cells and tracheids. Additionally, there were noticeable differences in the physical and chemical properties of the cell wall [72]. The correlation between the moisture content of Japanese cedar (*Cryptomeria japonica*) and the microcracks was employed by an enhanced confocal laser scanning electron microscope. The findings revealed that microcracks started to form near the fiber saturation point (28.9%), and cracks attained their maximum value as moisture content reached 9.9%, and then gradually closed with further moisture depletion. The cracks were first observed between the tracheid and the ray parenchyma cells in latewood. As the moisture content decreased, the crack propagated along the ray parenchyma cell towards both ends, and the crack tip ended at the boundary of the next growth ring [73,74]. As shown in Figure 5, Gao et al. conducted a study investigating the cracking of Masson pine (*Pinus massoniana*) tracheids during drying. Results showed that cracks initially manifested in the intercellular layer between the ray tissue and the tracheid, as well as between the ray cells [22].

## 6. Summary and Prospect

Drying stress, primarily induced by the anisotropy of drying shrinkage and the moisture content gradient during wood processing, is the main contributor to wood deformation and cracking under moisture loss. The drying stress is principally associated with the inherent drying shrinkage properties of the wood, the thickness of the board, and surrounding temperature and humidity conditions. The disparity between tangential and radial drying shrinkage of wood is determined by the inherent characteristics of wood. This disparity is an inevitable part of the drying process, especially in wood cross-section discs drying, which have to handle the impacts of this in processing and in applications. However, the moisture content gradient during the drying process can be modulated by controlling the surrounding temperature and humidity conditions. Mitigating the moisture content gradient in wood throughout the drying process serves as an effective strategy to minimize wood deformation and cracking. Currently, numerous studies focus on the differences of tangential and radial shrinkage and the moisture gradient during wood drying, but a quantitative depiction of the relationship between drying stresses and wood cracking. Consequently, future research must emphasize the accurate detection of drying stresses within the process of wood drying, in conjunction with quantifying the correlation between drying stress and wood deformation and cracking. Except for the drying process, MC change can also deform boards, leading to cupping and other deformation in service. In addition, there are some treatment method such as microwave technology, is a useful way to relief of growth and drying stresses in rapid drying of hardwoods [75,76].

Identifying the relationship between the transverse mechanical properties of wood and its moisture content is key to understanding the drying cracking during the moisture discharge process. Therefore, future studies should explore variations in tangential tensile strength in different moisture conditions and within specific areas of the wood, such as earlywood and latewood, or heartwood and sapwood. The macroscopic mechanical properties of wood are largely determined by its microscopic structure. This is primarily observed in the dynamic relationship between the degree of cellulose crystallinity in the wood and moisture, i.e., the crystalline and amorphous regions of the cellulose microfibrils are changed with variation moisture content. Current studies have extensively researched the relationship between the mechanical properties of wood cell walls and moisture, yet studies evaluating the impact of the main chemical components of wood on its mechanical properties remain scarce. Emphasizing the effect of moisture content changes on wood mechanical properties at the cell wall level and molecular level will be a pivotal aspect of future research. This focus is critical for elucidating the mechanisms of wood deformation and cracking in response to moisture loss.

The transverse tensile strength of wood is crucial in determining its deformation and cracking behavior under moisture loss. Research into the mechanical properties of wood in transverse direction, particularly the tangential tensile strength, has been comparatively limited. Identifying the relationship between the transverse mechanical properties of wood and its moisture content is key to understanding the drying cracking during the moisture loss. Therefore, future studies should explore variations in tangential tensile strength in different moisture conditions and within specific areas of the wood, such as earlywood and latewood.

## Data Availability

Not applicable.
